# *TMEM187-IRAK1* Polymorphisms Associated with Rheumatoid Arthritis Susceptibility in Tunisian and French Female Populations: Influence of Geographic Origin

**DOI:** 10.1155/2017/4915950

**Published:** 2017-02-08

**Authors:** Olfa Khalifa, Nathalie Balandraud, Nathalie Lambert, Isabelle Auger, Jean Roudier, Audrey Sénéchal, David Geneviève, Christophe Picard, Gérard Lefranc, Isabelle Touitou, Bakridine M'Madi Mrenda, Cécilia Benedito, Etienne Pardoux, Anne-Laure Gagez, Yves-Marie Pers, Christian Jorgensen, Touhami Mahjoub, Florence Apparailly

**Affiliations:** ^1^Inserm, U 1183, Institute for Regenerative Medicine and Biotherapies, CHU Saint Eloi, 80 Avenue Augustin Fliche, 34295 Montpellier Cedex 5, France; ^2^University of Montpellier, Boulevard Henri IV, 34090 Montpellier, France; ^3^Inserm UMRs 1097, Aix-Marseille University, Marseille, France; ^4^Rheumatology Department, APHM, Marseille, France; ^5^Inserm, U1051, University Hospital Saint Eloi, Institute for Neurosciences of Montpellier, Montpellier, France; ^6^Department of Clinical Genetics, University Hospital of Montpellier, Montpellier, France; ^7^Aix-Marseille University, CNRS, EFS, ADES UMR 7268, 13916 Marseille, France; ^8^Laboratoire d'Immunogénétique Moléculaire, UPR 1142 CNRS, Institute of Human Genetics, Montpellier, France; ^9^Department of Molecular Genetics, University Hospital of Montpellier, France; ^10^Aix-Marseille University, CNRS, Centrale Marseille, I2M, UMR 7373, 13453 Marseille, France; ^11^CNRS UMR 5235, Université de Montpellier, Montpellier, France; ^12^Clinical Department for Osteoarticular Diseases and Biotherapy, University Hospital Lapeyronie, 34295 Montpellier, France; ^13^Laboratory of Human Genome and Multifactorial Diseases, Faculty of Pharmacy, University of Monastir, Monastir, Tunisia

## Abstract

Polymorphisms have been identified in the Xq28 locus as risk loci for rheumatoid arthritis (RA). Here, we investigated the association between three polymorphisms in the Xq28 region containing* TMEM187* and* IRAK1* (rs13397, rs1059703, and rs1059702) in two unstudied populations: Tunisian and French. The rs13397 G and rs1059703 T major alleles were significantly increased in RA patients (*n* = 408) compared with age-matched controls (*n* = 471) in both Tunisian and French women. These results were confirmed by a meta-analysis replication study including two independent Greek and Korean cohorts. The rs1059702 C major allele was significantly associated with RA, only with French women. In the French population, the GTC haplotype displayed a protective effect against RA, while the ATC, GCC, and GTT haplotypes conferred significant risk for RA. No association for these haplotypes was found in the Tunisian population. Our results replicated for the first time the association of the three Xq28 polymorphisms with RA risk in Tunisian and French populations and suggested that RA susceptibility is associated with* TMEM187-IRAK1* polymorphisms in women. Our data further support the involvement of X chromosome in RA susceptibility and evidence ethnicities differences that might be explained by differences in the frequencies of SE HLA-DRB1 alleles between both populations.

## 1. Introduction

Rheumatoid arthritis (RA) is a systemic autoimmune disease. It is characterized by chronic destructive inflammation in synovial joints. The prevalence of RA is about 1% in the adult European population and is three times more common in women than in men [1–4]. The aetiology of this complex disease is still poorly understood but is considered as a result of interaction between susceptibility genes and environmental factors [5]. The genetic contribution to RA has been estimated to be about 50–60% [6], with the HLA (Human leukocyte antigen) classes II molecules remaining the most powerful known genetic factor [7]. There is extensive evidence for the association between certain HLA-DRB1 alleles that contain a conserved sequence of five amino acids (Q/RK/RRAA) in the third hypervariable region of the DR*β*1 chain, the so-called shared epitope (SE), and RA susceptibility and severity [8–10].

Genome-wide association studies (GWAS) and high-density array Immunochip studies for single nucleotide polymorphisms (SNPs) genotyping of populations have identified over 101 RA risk loci involved among individuals of European and Asian ancestry [11–15]. Among these,* IRAK1* (interleukin 1 receptor associated kinase) was the first X chromosome locus reported as associated with RA susceptibility and is thus of importance given the female predominance of the disease.* IRAK1* is a serine-threonine protein kinase and an essential component of the toll/interleukin 1 receptor (TIR) signaling pathway involved in the pathogen-mediated inflammation [16]. Interestingly, the* IRAK1* gene is located on the Xq28 region that harbours several SNPs that have also been associated with susceptibility to autoimmune diseases. A recent case-control study investigated numerous SNPs located on the Xq28 locus and identified rs1059703 and rs1059702, encoding for pSer532Leu and pPhe196Ser, as two* IRAK1 *SNPs most significantly associated with RA susceptibility in Korean families [17]. These authors also showed that the major haplotype (rs1059702 T and rs1059703 C) was associated with increased* IRAK1 *activity. Of note, upstream* IRAK1 *and within the Xq28 risk locus, Eyre et al. reported the SNP rs13397 associated with RA risk among individuals of northern European ancestry [13]. This polymorphism is located within the* TMEM187 *gene, which encodes a transmembrane protein of unknown function.

The relationship between these three polymorphisms on the Xq28 region and RA risk in different population remains unaddressed. Interestingly, a clear separation of different ethnic and regional populations was evidenced when considering northern and southern Europe groups separately in genetic studies, suggesting the existence of European population genetic substructures [18]. In addition, there are also clear differences between northern and southern Europe for the main genetic risk factor for RA, HLA-DRB1^*∗*^04:04, and DRB1^*∗*^04:01 genotypes being the most frequent in northern populations, while HLA-DRB1^*∗*^10:01 and DRB1^*∗*^04:05 alleles are more frequent in southern Europe, as well as in Maghreb [19, 20]. Therefore, the present study aimed at investigating the association between critical polymorphisms in Xq28, from rs13397 (A/G) through rs1059703 (C/T) and rs1059702 (C/T) in the* TMEM187 *and* IRAK1* locus, respectively, with risk for RA in two not previously studied populations displaying different genetic background: Tunisian and French. We also examined genetic differences for the HLA-DRB1 alleles in both RA populations. A meta-analysis including two other independent cohorts was then performed to test the overall effect of these* TMEM187-IRAK1 *polymorphisms on RA.

## 2. Patients and Methods

### 2.1. Study Subjects

For RA patients and healthy controls, informed consents were provided in accordance with national procedures. French studies were approved by local human ethical committees: sample collection and analysis (DC-2008-327) were approved by the “Cellule Bioéthique, Direction Générale pour la Recherche et l'Innovation, Ministère de l'Enseignement Supérieur et de la Recherche” (Ministry Bioethics Unit) and by Comité de Protection des Personnes Sud Méditerrannée IV (ID RCB 2008-A01087-48). Blood samples were collected from a total of 879 unrelated female participants: 408 patients with RA and 471 controls from Tunisia and France. Detailed size of Tunisian and French cohort is shown in Table 1. RA patients fulfilled the 2010 ACR/EULAR classification criteria for RA [21]. The clinical examination consisted of questionnaire and a physical examination: age, geographical origin, family history, body mass index, smoking habits, the presence of hypertension, diabetes, levels of physical activity, and alcohol consumption. The 471 controls matched with age were recruited from Voluntary Bone Marrow Donor (VBMD). Less than 1% of healthy VBMD women come from Maghreb. All patients were positive for anti-citrullinated peptide antibodies (ACPA) as determined by commercially available ELISA kits (Orgentec®, Hamburg, Germany) and Euroimmun® for Tunisian cases and CCP2 enzyme-linked immuno sorbent assay (ELISA) (Immunoscan RA, Euro-Diagnostica, Arnhem, Netherlands) for French cases.

### 2.2. Genotyping

Selection for Xq28 polymorphisms from previously reported SNPs was conducted using the following criteria: (a) the minor allele frequency (MAF) was >0.05 in the Caucasian population, according to the international HapMap project databank (http://www.hapmap.org) and to the NCBI SNP database (https://www.ncbi.nlm.nih.gov), (b) it was a tagging SNP, and (c) it has been reported to be associated with RA or other autoimmune diseases in different populations or other types of studies as GWAS. Genomic DNA was extracted from total blood leukocytes using standard methods including proteinase K digestion, followed by phenol–chloroform extraction and ethanol precipitation. Finally, all subjects were genotyped for three SNPs (rs1059702 and rs1059703 and rs13397) using direct PCR sequencing with the BigDye Terminator v3.1 Cycle Sequencing Kit (Applied Biosystems) and an Applied Biosystems (ABI) 3130xL genetic analyzer (Applied Bio Systems, Foster City, CA, USA). PCR reactions were performed in a Bio-Rad thermal cycler using Taq Polymerase (Prim 5 by Fisher Scientific) with initial denaturing conditions at 96°C for 5 min, followed by 30 cycles of 96°C for 30 s, 64°C for 30 s, abd 72°C for 45 s and a final extension of 72°C for 10 min.

### 2.3. HLA-DRB1 Genotyping

Genomic DNA from whole blood was extracted as described in the subsection “Genotyping.” HLA-DRB1 typing was carried out according to the manufacturer's specification for LAB type SSO HD (One Lambda Inc., USA) and the retrieved output was analyzed by HLA Fusion v 1.2.1 software (One lambda Inc., USA) for allele information. The following alleles or group of alleles were genotyped: HLA-DRB1^*∗*^01, HLA-DRB1^*∗*^01:03, HLA-DRB1^*∗*^03, HLA-DRB1^*∗*^04:01, HLA-DRB1^*∗*^04:02, HLA-DRB1^*∗*^04:03, HLA-DRB1^*∗*^04:04, HLA-DRB1^*∗*^04:05, HLA-DRB1^*∗*^04:06, HLA-DRB1^*∗*^04:07, HLA-DRB1^*∗*^04:08, HLA-DRB1^*∗*^04:11, HLA-DRB1^*∗*^07, HLA-DRB1^*∗*^08, HLA-DRB1^*∗*^09, HLA-DRB1^*∗*^1001, HLA-DRB1^*∗*^11, HLA-DRB1^*∗*^12, HLA-DRB1^*∗*^13, HLA-DRB1^*∗*^14, HLA-DRB1^*∗*^15, and HLA-DRB1^*∗*^16 (Table 2).

### 2.4. Meta-Analysis Study

A literature search using PubMed, Science Direct, and Web of Science databases was conducted to identify publications that examine the association of* TMEM187* rs13397,* IRAK1* rs1059703,* IRAK1* rs1059702, and* IRAK1* rs3027898 polymorphisms with RA. Our research strategy was based on a combination of the following keywords: variation, variant, polymorphism, SNP, Rheumatoid arthritis, RA,* TMEM187, IRAK1*, rs13397, rs1059703, rs1059702, and rs3027898. We also reviewed the references mentioned in the identified articles for any additional relevant studies. Association studies included in our meta-analyses had to meet the following criteria: (1) evaluation of association between* TMEM187* rs13397,* IRAK1 *rs1059703,* IRAK1* rs1059702, or* IRAK1* rs3027898 polymorphisms and the susceptibility to RA, (2) use of case-control design, and (3) inclusion of available genotype frequencies or sufficient information for calculation. The following information was extracted from the selected studies: first author, years of publication, characteristics of cases and controls (mean age, distribution of gender, and ethnicity), number of cases and controls, and genotype/allele frequency information. The strength of the association between the three polymorphisms and RA was measured by pooled OR with 95% CI. The *Z* test was used to determine the significance (*p* < 0.05) of the pooled OR. The heterogeneity between studies was checked by *Q* test and *I*^2^ statistics. We used a fixed effect model when *p* value of the *Q* test was less than 0.10 or *I*^2^ was equal to or less than 50%; otherwise, random effect model was used. Finally this meta-analysis was performed using Review Manager 5.3 software (Cochrane Collaboration, London, UK).

### 2.5. Statistical Methods

In this study, the Bayesian statistical method was used to evaluate the confidence intervals (called in the Bayesian framework credibility intervals) and to compute the *p* values associated with our tests, as in [20]. Indeed, the Bayesian statistical methods are more appropriate in case of small samples and the “approximate value” of the variance of the logarithm of the Odds Ratio. In the Bayesian framework, the unknown parameter is considered as the realization of a random variable, which forces us to choose an a priori probability distribution for that random variable. We chose an “a priori” distribution for a pair of frequencies and decided that the two random variables are independent, each with the probability density equal to 1 on the interval (0.1). It is fair to say that our prior is uninformative: it is the uniform distribution on the interval (0.1). The “a posteriori” distribution of each parameter is easily shown from the Bayes formula to be a Beta distribution. More precisely, if while observing *n* realizations of 1 s and 0 s, we get *n* 1 s and *n*′ 0 s, then the a posteriori distribution of the proportion is the Beta (*n* + 1, *n*′ + 1) probability distribution. Since the two samples come from completely unrelated sources, it is fair to assume that the two proportions are conditionally independent, given the data. Consequently a posteriori distribution of the pair of parameters (corresponding to the proportions of the given allele among the patients and among the control individuals) is the product of two Beta distributions whose density is explicitly known. Our method involves simulating one million realizations of that pair of random variables, from which we deduce one million realizations of the Odds Ratio. The *p* value of the test is then obtained by looking at which proportion of those values is smaller (resp., larger) than 1; this gives us *t*. Finally we want to discuss the issue of multiple testing. We have used our Bayesian test method 6 times (Table 5) where the probability of an error among six successive tests is not controlled by the probability of an error in each single test. The *p* values were corrected for multiple comparisons using the Bonferroni correction in Table 2. PLINK 1.7 version software was used for analysis of association between haplotypes and disease, and Haploview 4.2 software was used for LD analysis. All haplotypes with frequencies less than 5% were ignored in analysis. Distributions of data were tested with the Shapiro-Wilk test. Chi^2^ or Fisher test was used for categorical data; Yates correction was performed. For numerical data, comparisons of medians were performed using Mann–Whitney or Student's *t*-tests. For correlation study, statistical analyses were performed at the conventional two-tailed *α* level of 0.05 using R software version 3.0.2.10. Odds ratios (OR) with 95% confidence interval (CI) were also calculated. A *p* value less than 0.05 was considered statistically significant.

## 3. Results

### 3.1. Demographics and Phenotypes of the Study Subjects

Characteristics of the 408 ACPA (anti-citrullinated protein antibody) positive RA and 471 controls from Tunisia and South Eastern France (Marseille and Montpellier) are shown in Table 1. All samples were females. The average age was 52 years for the Tunisian RA and 63 years for French RA women. Age was not significantly different between patients and control females. There were more presently smokers in controls than in patients group.

### 3.2. Frequency of HLA-DRB1 Alleles and SE

Allelic frequencies of the HLA-DRB1 alleles in Tunisian and French RA patients are presented in Table 2. Due to the size of cohorts, we applied Bayesian statistical method to define susceptibility or protective genotypes and calculate accurate confidence intervals for the associated Odds Ratios. HLA-DRB1^*∗*^01, HLA-DRB1^*∗*^03, HLA-DRB1^*∗*^04:01, HLA-DRB1^*∗*^04:04, HLA-DRB1^*∗*^04:05, HLA-DRB1^*∗*^04:06, HLA-DRB1^*∗*^10, HLA-DRB1^*∗*^13, HLA-DRB1^*∗*^15, and HLA-DRB1^*∗*^16 showed statistical differences between the French and the Tunisian RA women. No statistical differences were found for HLA-DRB1^*∗*^01:03, HLA-DRB1^*∗*^04:02, HLA-DRB1^*∗*^04:03, HLA-DRB1^*∗*^04:07, HLA-DRB1^*∗*^04:08, HLA-DRB1^*∗*^04:11, HLA-DRB1^*∗*^07, HLA-DRB1^*∗*^08, HLA-DRB1^*∗*^09, HLA-DRB1^*∗*^11, HLA-DRB1^*∗*^12, and HLA-DRB1^*∗*^14 between both populations. The RA associated HLA-DRB1 alleles that were significantly more present in the Tunisian samples compared to those in the French samples were, by order of Odds Ratios, HLA-DRB1^*∗*^04:05 (OR = 2.15), HLA-DRB1^*∗*^13 (OR = 2.39), HLA-DRB1^*∗*^10 (OR = 3.65), HLA-DRB1^*∗*^03 (OR = 4.17), and HLA-DRB1^*∗*^04:06 (OR = +∞). Similarly, RA associated HLA-DRB1 alleles that were significantly more observed in the French than in the Tunisian samples were, by order of Odds Ratios, HLA-DRB1^*∗*^16 (OR = 0.09), HLA-DRB1^*∗*^04:01 (OR = 0.26), HLA-DRB1^*∗*^01 (OR = 0.31), HLA-DRB1^*∗*^04:04 (OR = 0.32), and HLA-DRB1^*∗*^15 (OR = 0.45). Three HLA-DRB1 alleles (DRB1^*∗*^04:05, DRB1^*∗*^12:XX, and DRB1^*∗*^15:XX) did not show significant differences between Tunisian and French RA women after the Bonferroni correction.

Two (HLA-DRB1^*∗*^04:05 and HLA-DRB1^*∗*^10) out of seven shared SE alleles were overrepresented in the Tunisian RA population, and three SE alleles (HLA-DRB1^*∗*^01, HLA-DRB1^*∗*^04:01, and HLA-DRB1^*∗*^04:04) were overrepresented in the French population. SE single dose showed similar frequency in both Tunisian and French RA women (50% and 57.80%, resp.), while French RA population displayed higher SE double dose than Tunisian RA population (20.34% and 9.09%, resp.) (Table 3).

### 3.3. Association between Three* TMEM187-IRAK1* Polymorphisms and RA Susceptibility in Women Samples from Tunisian and French Populations

Genotype distributions of all three investigated SNPs were in Hardy-Weinberg equilibrium in both patient and control groups (*χ*^2^ < 3.84). Among the genotype frequencies of the three SNPs analyzed using the dominant modelling, the rs13397 and rs1059703 demonstrated a significant association with RA susceptibility in both populations. The rs13397 G allele is present in 75% of RA Tunisian patients and in 69% RA French (Table 4). The frequency of the rs13397 G major allele was significantly lower in RA patients than in controls in both Tunisian (OR 0.48 [95% CI 0.12–0.73], *p* = 0.0025) and French (OR = 0.45 [95% CI 0.21–0.59], *p* < 0.001) women populations. The rs1059703 T major allele was significantly decreased in patients compared with controls in both Tunisian (OR = 0.46 [95% CI 0.13–0.71], *p* < 0.001) and French (OR = 0.36 [95% CI 0.15–0.47], *p* < 0.001) women populations. The SNP rs1059702 C showed a significant association with RA in French patients as compared with control subjects (OR 1.97 [95% CI 1.22–11.52], *p* < 0.001), but none in the Tunisian population. Of note, no correlation was found between polymorphisms and the SE (data not shown).

### 3.4. Linkage Disequilibrium of* TMEM187-IRAK1* Locus with RA in the Samples of the Tunisian and French Populations

**Figure 1:**
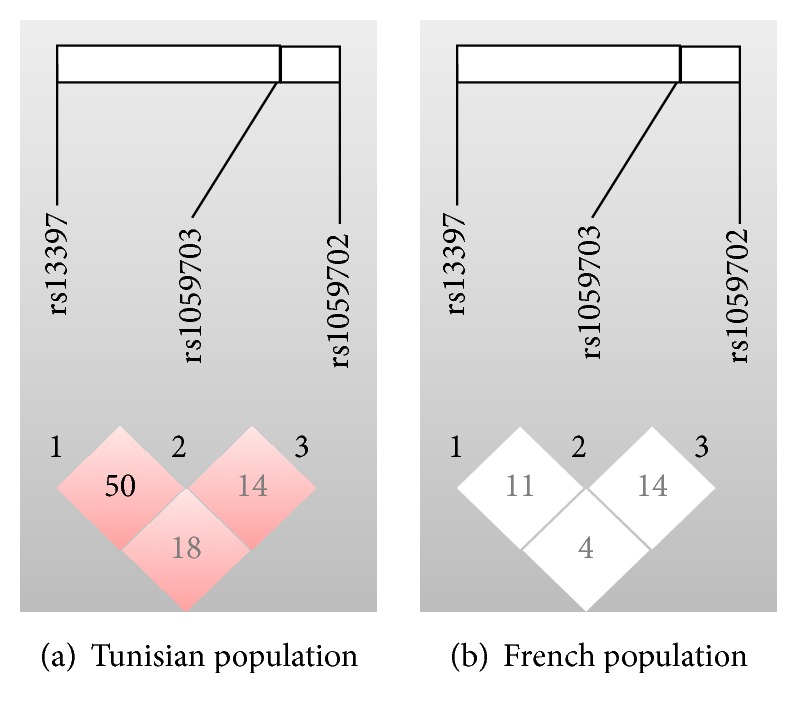
Linkage disequilibrium (LD) between* TMEM187-IRAK1* SNPs. Correlations (*r*^2^ values) among all 3 single nucleotide polymorphisms (SNPs) located in the* TMEM187-IRAK1* locus are indicated in the diamonds for the Tunisian (a) and French (b) populations. Haploview 4.2 software was used for LD analyses.

To determine the extent of linkage disequilibrium (LD) among the three polymorphisms enclosed in the* TMEM187-IRAK1* locus in both Tunisian and French populations, standardized LD coefficient *r*^2^ was calculated for all pairs of the three polymorphisms. Our study showed that the* IRAK1 *SNPs rs1059703 and rs1059702 did not belong to the same linkage disequilibrium locus, both in Tunisian and French populations (Figure 1). We also showed that rs13397 and rs1059703 polymorphisms were in moderate linkage disequilibrium (*r*^2^ = 0.5) in the Tunisian population (Figure 1(a)), but not in the French population (Figure 1(b), *r*^2^ = 0.11).

### 3.5. Haplotype Analysis

To examine the combined effect of three variants within the* TMEM187-IRAK1* locus, the haplotype frequencies defined by the three SNPs rs13397, rs1059703, and rs1059702 were analyzed. Among the potential haplotypes, four common haplotypes were identified in our populations with frequencies greater than 5% in both cases and controls (Table 6).

Results of the analysis suggested that the GTC haplotype, which carries the rs13397 G variant, displayed a protective effect against RA in our sample of the French population (OR = 0.23 (95% CI) [0.16–0.33], *p* < 0.001) while the ATC, GCC, and GTT haplotypes conferred significant risk for RA in the same population. French women with the ATC, GCC, or GTT haplotypes had 3.97-, 2.39-, and 2.05-fold higher risk of having RA compared to controls. In contrast, association between all 4 detected haplotypes and disease was not significant in the sample of the Tunisian women. Overall, these data further support the involvement of the Xq28 locus in RA susceptibility in the French population and underscore ethnical differences.

### 3.6. Meta-Analysis Study

**Figure 2:**
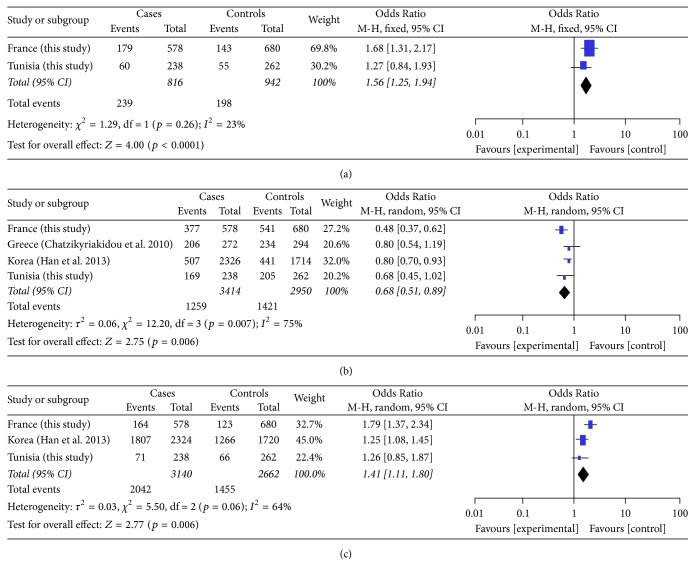
Forest plot of the association between* TMEM187* rs13397 (a),* IRAK1* rs1059703 (b), and* IRAK1* rs1059702 (c) polymorphisms and RA. Meta-analyses were performed for the rs13397 (a), rs1059703 (b), and rs1059702 (c) polymorphisms, respectively, using Review Manager 5.3 software.

A meta-analysis was performed and included datasets from 2076 RA cases and 2112 healthy controls. To carry out the meta-analysis, we combined our findings with three independent studies that fit the inclusion criteria and included a total of 1668 RA patients and 1641 ethnically matched healthy controls from Greece and Korea [17, 22, 23]. The samples size of these studies varied from 119 to 1318 cases and from 131 to 1016 controls. The characteristics of all studies were summarized in Table 7. Meta-analysis results showed a significant association between* TMEM187 *rs13397 (OR = 1.56, 95% CI = 1.25–1.94, and *p* value < 0.0001) and* IRAK1* rs1059702 (OR = 1.41, 95% CI = 1.11–1.80, and *p* value = 0.006) polymorphisms and susceptibility to RA (Figures 2(a) and 2(c)). The* IRAK1* rs1059703 polymorphism showed a significant protective effect in RA (OR = 0.68, 95% CI = 0.51–0.89, and *p* value = 0.006) (Figure 2(b)). The* IRAK1* rs3027898 polymorphism that was available in the three independent studies from Korea, China, and Greece showed no significant association with RA risk (OR = 0.96, 95% CI = 0.67–1.39, and *p* value = 0.83).

## 4. Discussion

In the present study, we aimed at investigating the association between three critical polymorphisms in the* TMEM187-IRAK1 *locus and risk for ACPA positive RA female patients in two not previously studied populations: Tunisian and French. We found that the rs13397 A and rs1059703 C risk alleles were significantly associated with RA susceptibility for both populations. The rs1059702 T allele was only found associated with risk for RA in the French sample. Four haplotypes were detected in both populations. In the French population, the major GTC haplotype displayed a protective effect against RA, whereas the GCC, GTT, and ATC haplotypes conferred significant increased risk for RA. No association for these haplotypes was however found in the Tunisian population. Finally, a strong genetic association of RA susceptibility exists for the rs13397/rs1059703 block only in women Tunisian subjects.

Among the many loci that have been identified as associated with RA risk using either candidate gene association studies or GWAS,* IRAK1 *is the only gene located on the X chromosome. The* IRAK1 *gene encodes the interleukin 1 receptor-associated kinase 1, a serine/threonine kinase that mediates TLRs and IL-1 receptor signaling. Upon receptor stimulation,* IRAK1* is recruited, phosphorylated, and responsible for TLR- and IL1-induced activation of the NF-*κ*B signaling cascade, thus playing a critical role in the early inflammatory response [24]. The* IRAK1 *gene is located within the Xq28 locus that contains several polymorphisms associated with increased susceptibility to autoimmunity, including systemic lupus erythematous [25], systemic sclerosis [26, 27], and RA [13, 17, 22, 23]. Altogether, these studies demonstrated that the Xq28 locus is a shared locus for several complex diseases, thereby highlighting its role in inflammatory disease susceptibility. Recently, two studies devoted attention to the* TMEM187-IRAK1 *locus and identified two SNPs in a Korean population (rs1059702 and rs1059703) [17] and one SNP (rs13397) in a large cohort of northern European ancestry [13], respectively, associated with increased disease susceptibility. In an attempt to investigate new, not previously studied populations and to replicate the above studies, we examined the distribution of these three SNPs and found statistical differences between RA patients and controls for both Tunisian and French populations. Although risk alleles for both rs1059702 and rs1059703 were different, they were also associated with risk for RA in both Tunisian and French populations. Indeed, the rs1059702 T and rs1059703 C polymorphisms were in strong association with RA in Korean women while we found the rs1059702 C and rs1059703 T alleles in our cohorts. In agreement with the study of Eyre et al. [13], we found the minor rs13397 A allele to be the risk allele in both the French and Tunisian cohorts. Earlier work performed on a small Greek cohort found no association between the rs1059703 and RA [22]. These differences might have several explanations, including the sample size of cohorts used, the Bayesian statistical method being better suited than the SPSS statistical package to decide about susceptibility in small samples, the classification criteria used to select RA patients (ACR 1987 versus 2010 ACR/EULAR that discriminate or not between ACPA positive RA patients), and different ethnic and regional populations.

Concerning the haplotype and LD studies, we are the first to study the combination of LD and haplotypes of these three SNPs with RA. We found that, with the exception of rs13397 and rs1059703 polymorphisms, which were in moderate linkage disequilibrium in the Tunisian population (*r*^2^ = 0.5), other polymorphisms were not in linkage disequilibrium in both population. This finding seems to be in contradiction with the Korean study that previously reported a linkage disequilibrium block on Xq28 enclosing both rs1059702 and rs1059703 [17]. Discrepancies between Korean, Tunisian, and French populations might be due to differences in genetic background. Interestingly, a comparative study reported that none of the susceptibility loci identified by GWAS in Caucasian patients with RA contributed significantly to disease in Koreans [28].

We found differences between French and Tunisian populations for association of SNP haplotypes in the* TMEM187-IRAK1 *locus with RA susceptibility. The CTG haplotype displayed a protective effect against RA, while the ATC, GCC, and GTT haplotypes conferred significant susceptibility to RA, in French women. All four haplotypes showed no association in the Tunisian women sample. In both Tunisian and French cohorts, we found the GTC common haplotype in 57 and 68% of the control populations, respectively. This GTC haplotype carries the major alleles for the three SNPs rs13397 G, rs1059703 T, and rs1059702 C.

Although alleles and haplotypes identified among Tunisian and French subjects display differences with previous studies performed with Korean and northern European cohorts, our present work confirms significant association of the three SNPs with RA susceptibility. The minor discrepancy found for the rs1059702 in the Tunisian RA women population might be due to the size of the cohort. Indeed, pooled results for the three different ethnical groups including Tunisian confirm a significant association between rs1059702 and risk for RA.

Our meta-analysis including a larger case-control data set of two independent studies from Korea and Greece in addition to our data sets confirmed that* TMEM187* rs13397,* IRAK1* rs1059702, and* IRAK1* rs1059703 are associated with RA. Unfortunately, data sets on the* TMEM187* rs13397 from populations of European ancestry [13] were not available for inclusion in our meta-analysis. The* IRAK1* rs3027898 that was not investigated in our study but available in the three independent studies from Korea, China, and Greece showed no significant association in the meta-analysis (data not shown). To the best of our knowledge, this is the first meta-analysis study on the association between RA and these four polymorphisms in* TMEM187-IRAK1*.

Many studies have documented that one of the key genetic elements closely linked to RA susceptibility is the HLA complex locus located in the chromosome 6 [15]. Our present study evaluated the frequency of HLA-DRB1 alleles in Tunisian and French RA women, all ACPA positive, and confirmed that both populations belonging to different ethnical groups do not share the same HLA susceptibility alleles. As expected for the French population, we find the usual association between RA and HLA-DRB1^*∗*^01, HLA-DRB1^*∗*^04:01, and HLA-DRB1^*∗*^04:04 [20]. We show that two SE alleles, HLA-DRB1^*∗*^04:05 and HLA-DRB1^*∗*^10, are overrepresented in the RA Tunisian population. This is in agreement with a previous report showing their association with RA susceptibility in Tunisia [19]. The HLA-DRB1^*∗*^04 allele has also been reported in RA populations from Morocco [29], Saudi Arabia [30], and Turkey [31]. The HLA-DRB1^*∗*^10 allele has been reported to be associated with RA in Saudi Arabia [30], Italy [32], Spain [33], Turkey [34], and Asian Indian [35] populations.

A meta-analysis performed in Latin America RA population (Colombia, Peru, Brazil, Argentina, Mexico, and Chili) showed that HLA-DRB1^*∗*^04:04 allele is associated with disease risk [36]. Moreover, this whole genome scan study also shows that the second most consistently linked region to RA is the Xq28 locus. The absence of the HLA-DRB1^*∗*^01:03, HLA-DRB1^*∗*^04:07, and HLA-DRB1^*∗*^12 alleles in our RA Tunisian population sample is in accordance with the protective effect suggested by previous study [19]. Considering the observed differences between ethnical groups for HLA-DRB1, our findings suggest that the association of the* TMEM187-IRAK1* locus with RA risk may play a role independently of the major genetic risk locus for RA.

## 5. Conclusion

In conclusion, the present study illustrates the involvement of the Xq28 locus in RA susceptibility in both Tunisian and French populations with ethnical differences. Finally, the meta-analysis combining our findings with two independent studies from Greece and Korea further supports the fact that the three polymorphisms are important in the susceptibility for RA. Although a growing number of studies suggest the contribution of the X chromosome to RA, as to many other autoimmune diseases, future works shall be performed to compare female and male RA populations to identify the exact contribution of this locus, with a focus on TMEM187-IRAK1 polymorphisms. Furthermore, functional studies on the impact of these polymorphisms on both gene expressions in RA populations might help defining new therapeutic perspectives in RA.

## Figures and Tables

**Table 1:** Characteristics of female samples from Tunisian and French cohort.

Characteristic	Tunisian population		French population	
	Controls	RA patients	Controls	RA patients
Size of the cohort	131	119	340	289
Age (mean ± SD years)	56.65 ± 17.37	52.16 ± 13.61	46.24 ± 8.92	63.15 ± 20.71
Disease duration (years)	NA	15.3 ± 5.6	NA	14.8 ± 6.9
Positive ACPA, *n* (%)	NA	119 (100)	NA	289 (100)
Positive RF, *n* (%)	NA	98 (74.7)	NA	200 (69.20)
C-reactive protein (mg/L)	NA	12.2 ± 22.7	NA	13.6 ± 21.8
DAS28	NA	6.3 ± 1.2	NA	5.2 ± 1.4
Smoking status%	66	55	76	47

**Table 2:** Shared epitope frequency and *HLA-DRB1* alleles frequency in Tunisian and French RA.

Alleles HLA-DRB1	Tunisian RA Allele number (*N* = 264)	Tunisian allele frequency (%)	French RA Allele number (*N* = 346)	French allele frequency (%)	OR [95% CI]	*p* value	*p*value^*∗*^
^#^DRB1^*∗*^01:XX	16	6.06	59	22.35	0.31 [0–0.51]	1.6*E*^−05^	35.2*E*^−05^
DRB1^*∗*^01:03	0	0.00	1	0.38	0 [0–4.55]	0.32	NS
DRB1^*∗*^03:XX	42	15.91	15	5.68	4.17 [2.48–+∞]	1*E*^−06^	22*E*^−06^
^#^DRB1^*∗*^04:01	11	4.17	48	18.18	0.26 [0–0.47]	1.5*E*^−05^	33*E*^−05^
DRB1^*∗*^04:02	1	0.38	1	0.38	1.31 [0.20–+∞]	0.39	NS
DRB1^*∗*^04:03	3	1.14	4	1.52	0.98 [0–3.23]	0.51	NS
^#^DRB1^*∗*^04:04	8	3.03	30	11.36	0.32 [0–0.64]	0.001	0.0022
^#^DRB1^*∗*^04:05	19	7.20	12	4.55	2.15 [1.16–+∞]	0.01	NS
DRB1^*∗*^04:06	6	2.27	0	0.00	+∞ [2.49–+∞]	0.002	0.044
DRB1^*∗*^04:07	0	0.00	1	0.38	0 [0–4.56]	0.32	NS
^#^DRB1^*∗*^04:08	3	1.14	9	3.41	0.43 [0–1.27]	0.11	NS
DRB1^*∗*^04:11	1	0.38	0	0.00	+∞ [0.37–+∞]	0.18	NS
DRB1^*∗*^07:XX	41	15.53	46	17.42	1.19 [0.81–+∞]	0.21	NS
DRB1^*∗*^08:XX	3	1.14	1	0.38	3.96 [0.68–+∞]	0.11	NS
DRB1^*∗*^09:XX	1	0.38	2	0.76	0.65 [0–3.98]	0.41	NS
^#^DRB1^*∗*^10:01	33	12.50	13	4.92	3.65 [2.09–+∞]	2.3*E*^−05^	50.6*E*^−05^
DRB1^*∗*^11:XX	19	7.20	25	9.47	0.99 [0–1.66]	0.5	NS
DRB1^*∗*^12:XX	0	0.00	5	1.89	0 [0–0.83]	0.03	NS
DRB1^*∗*^13:XX	40	15.15	24	9.09	2.39 [1.52–+∞]	0.0005	0.011
DRB1^*∗*^14:XX	4	1.52	4	1.52	1.31 [0.43–+∞]	0.33	NS
DRB1^*∗*^15:XX	12	4.55	33	12.50	0.45 [0–0.80]	0.009	NS
DRB1^*∗*^16:XX	1	0.38	13	4.92	0.09 [0–0.49]	0.002	0.044

^#^Shared epitope, XX means all known alleles, DRB1^*∗*^14: XX apart from DRB1^*∗*^14:02

^*∗*^ *p* value corrected with Bonferroni correction.

NS: not significant.

**Table 3:** Shared epitope single dose/double dose frequency in Tunisian and French RA women.

Shared epitope (SE)	Tunisian RA *N* = 132	Tunisian SE frequency (%)	French RA *N* = 173	French SE frequency (%)
SE single dose	66	50.00	100	57.80
SE double dose	12	9.09	35	20.34

Total	78	59.09	135	78.48

**Table 4:** *TMEM187-IRAK1* allelic frequencies in samples of RA and healthy controls from Tunisian and French female populations.

Gene/SNPs	Populations	Alleles	Controls (%)	RA cases (%)
*TMEM 187 rs13397*	Tunisian	G	207 (79)	178 (75)
		A	55 (21)	60 (25)
	French	G	537 (79)	399 (69)
		A	143 (21)	179 (31)

*IRAK 1 rs1059703*	Tunisian	C	57 (22)	69 (29)
		T	205 (78)	169 (71)
	French	C	139 (20)	201 (35)
		T	541 (80)	377 (65)

*IRAK 1 rs1059702*	Tunisian	C	196 (75)	167 (70)
		T	66 (25)	71 (30)
	French	C	557 (82)	414 (72)
		T	123 (18)	164 (28)

**Table 5:** *TMEM187-IRAK1* genotypes for samples of RA and healthy controls in Tunisian and French female populations.

Gene/SNPs	Population	Genotype	Controls (%)	RA cases (%)	OR (95% CI)	*p* values
*TMEM 187 rs13397*	Tunisian	GG	92 (70)	66 (53)	0.48 (0.12–0.73)	0.0025
		AG	23 (18)	46 (41)		
		AA	16 (12)	7 (6)		
	French	GG	220 (65)	131 (45)	0.45 (0.21–0.59)	1.0*e* − 6
		AG	97 (28)	137 (47)		
		AA	23 (7)	21 (8)		

*IRAK 1 rs1059703*	Tunisian	CC	15 (11)	9 (7)	0.46 (0.13–0.71)	0.0017
		CT	27 (21)	51 (43)		
		TT	89 (68)	59 (50)		
	French	CC	27 (8)	34 (12)	0.36 (0.15–0.47)	1.0*e* − 6
		CT	85 (25)	133 (46)		
		TT	228 (67)	122 (42)		

*IRAK 1 rs1059702*	Tunisian	CC	81 (62)	62 (52)	0.67 (0.18–1.02)	0.0608
		CT	34 (26)	43 (36)		
		TT	16 (12)	14 (12)		
	French	CC	237 (70)	157 (54)	0.52 (0.24–0.68)	3.7*e* − 5
		CT	83 (24)	100 (35)		
		TT	20 (6)	32 (11)		

^*∗*^ *p* values compared genotypes according to the dominant/recessive model.

**Table 6:** Association of SNP haplotypes in the *TMEM187-IRAK1* region with RA susceptibility.

Chromosome	Populations	Haplotypes	Frequencies		OR (95%CI)	*p* values	Effects
			Controls	Cases			
X	Tunisian	[ATC]	0.15	0.15	1.00 (0.63–1.59)	0.995	NA
	French		0.09	0.19	3.97 (2.57–6.13)	<0.001	Risk
	Tunisian	[GCC]	0.14	0.17	1.21 (0.79–1.89)	0.379	NA
	French		0.06	0.15	2.39 (1.52–3.77)	<0.001	Risk
	Tunisian	[GTT]	0.15	0.18	1.09 (0.70–1.71)	0.692	NA
	French		0.04	0.10	2.05 (1.16–3.62)	0.009	Risk
	Tunisian	[GTC]	0.57	0.50	0.77 (0.57–1.04)	0.083	NA
	French		0.68	0.39	0.23 (0.16–0.33)	<0.001	Protector

Order of SNPs: [rs13397, rs1059703, rs1059702]

NA: no association.

**Table 7:** Characteristics of all studies included in meta-analysis within the *TMEM187-IRAK1 *locus.

Study	Population	Group	Subjects	Women's sex%	Average age in years	Genotype																			
						*TMEM 187*					*IRAK 1*					*IRAK 1*					*IRAK 1*				
						*rs13397*					*rs1059703*					*rs1059702*					*rs3027898*				
						GG	AG	AA	G	A	CC	CT	TT	C	T	CC	CT	TT	C	T	AA	AC	CC	A	C
This study	Tunisia	Case	119	100%	52.16 ± 13.61	66	46	7	178	60	9	51	59	69	169	62	43	14	167	71	—	—	—	—	—
		Control	131	100%	56.65 ± 17.37	92	23	16	207	55	15	27	89	57	205	81	34	16	196	66	—	—	—	—	—
This study	French	Case	289	100%	63.15 ± 20.71	131	137	21	399	179	34	133	122	201	377	157	100	32	414	164	—	—	—	—	—
		Control	340	100%	46.24 ± 8.92	220	97	23	537	143	27	85	228	139	541	237	83	20	557	123	—	—	—	—	—
Chatzikyriakidou et al. (2010)	Greece	Case	136	80%	60.8 ± 12.7	—	—	—	—	—	7	52	77	66	206	—	—	—	—	—	71	45	20	187	85
		Control	147	78%	56.23 ± 5.13	—	—	—	—	—	7	46	94	60	234	—	—	—	—	—	91	47	9	229	65
Han et al. (2013)	Korea	Case	1,318	100%	51.8 ± 12.2	—	—	—	—	—	715	389	59	1819	507	62	393	707	517	1807	719	383	56	1821	495
		Control	1,016	100%	36.7 ± 12.5	—	—	—	—	—	468	337	52	1273	441	59	336	465	454	1266	478	321	50	1277	421
Zhang et al. (2013)	China	Case	214	71%	54.63 (±15.77)	—	—	—	—	—	—	—	—	—	—	—	—	—	—	—	28	42	141	98	324
		Control	478	71%	54.84 (±10.45)	—	—	—	—	—	—	—	—	—	—	—	—	—	—	—	337	103	35	173	777

## Abbreviations

ACPA: Anti-citrullinated protein antibody
ACR: American College of Rheumatology
AR: Androgen receptor
CCP2: Cyclic-citrullinated peptide
CD40L: Cluster of differentiation 40 ligand
CI: Confidence interval
ELISA: Enzyme-linked Immunosorbent assay
EULAR: European League against Rheumatism
*FOXP3*: Forkhead box P3
GWAS: Genome-wide association studies
HLA-DRB1: Human leukocyte antigen-DRB1
IL2RG: Interleukin-2 receptor gamma chain
*IRAK1*: Interleukin 1 receptor associated kinase 1
LD: Linkage disequilibrium
MAF: Minor allele frequency
NF-kB: Nuclear factor-kappa B
OR: Odds Ratio
PCR: Polymerase chain reaction
RA: Rheumatoid arthritis
SNP: Single nucleotide polymorphism
SE: Shared epitope
TLR: Toll-like receptors
*TMEM187*: Transmembrane protein 187
VBMD: Voluntary bone marrow donor.
